# Urinary Tract Infection as the Diagnosis for Admission Through the Emergency Department: Its Prevalence, Seasonality, Diagnostic Methods, and Diagnostic Decisions

**DOI:** 10.7759/cureus.27808

**Published:** 2022-08-09

**Authors:** Sarah Alrashid, Ramah Ashoor, Sahar Alruhaimi, Amirah Hamed, Shahad Alzahrani, Abdulla Al Sayyari

**Affiliations:** 1 Medicine, King Saud bin Abdulaziz University for Health Sciences, College of Medicine, Riyadh, SAU; 2 Division of Nephrology and Renal Transplantation, Department of Medicine, King Abdulaziz Medical City, Ministry of the National Guard Health Affairs, Riyadh, SAU

**Keywords:** prevalence, esbl, admission, seasonality, uti, er

## Abstract

Background

Urinary tract infections (UTIs) affect millions of people of all ages around the world. It constitutes one of the most common conditions encountered in emergency departments (EDs). In this study, we aimed to inquire into the prevalence of UTIs as the hospitalization primary diagnosis through the emergency department and to research the seasonal pattern, accuracy of the diagnostic methods used, and final diagnosis.

Methods

A retrospective cross-sectional study was undertaken that included all patients admitted with a primary diagnosis of UTIs through the ED over a four-month period (January, April, June, and September) in the emergency department of King Abdulaziz Medical City (KAMC) in Riyadh. The prevalence, diagnostics, and outcomes of UTIs were evaluated, and their association with seasonality was assessed after obtaining data from the Hospital Information System BestCare of King Abdullah National Guard Hospital. The variables that have been collected were analyzed using the Statistical Package for Social Sciences software version 26 (IBM Corp., Armonk, NY, USA).

Results

A total of 315 patients were admitted with a diagnosis of UTI. The prevalence of UTI among patients admitted through the ED was 10.5% with a significantly higher prevalence noted in January (13.3%) than in April (8.5%) or September (8.8%) (Fisher’s exact test: 0.009 and 0.01, respectively). As would be expected, the cohort was made up of elderly individuals with a mean age of 70.6 years, and the male/female ratio was 1:2. UTI symptoms including dysuria, frequency, urgency, rigors, and loin pain were noted in only 41% of cases or less, and urinalysis was the basis of making the diagnosis (87.9% had positive leukocyte esterase (LE) and 90.5% had positive urine WBC/HPF). Furthermore, 4.4% required urgent treatment, and 3.1% required intensive care unit (ICU) admission. Urine culture was negative in 30.8% of the cases (30.8% false positives among those admitted with UTI). The commonest organisms isolated were *Escherichia coli* (33%), *Klebsiella pneumoniae* (14.3%), and *Pseudomonas aeruginosa* (5.1%). The median length of hospital stay (LOS) was 3.5 days, and the Charlson Comorbidity Index (CCI) score was 5.7. The mean hemoglobin (Hb), creatinine, C-reactive protein (CRP), procalcitonin, and lactic acid were 108 gm/L, 131.3 umol/L, 38.3 mg/L, 0.28 ng/mL, and 2.07 mmol/L, respectively.

Conclusion

This research found that the prevalence of UTI cases as an admission diagnosis through the emergency department was high, despite some cultures being negative or contaminated, thus probably indicating an increase in the rates of false positives. The admission rate is linked to factors such as oxygen saturation and RDW, but this is not entirely understood. In addition, the study also displayed a seasonal pattern linked to the highest number of confirmed cases in January, while the lowest was in April.

## Introduction

Urinary tract infections (UTIs) are among the highly prevalent bacterial infections affecting millions of people worldwide. All age groups and both sexes are commonly affected [1]. Dysuria, urgency, and frequency are among the common symptoms of UTI. Female patients may also complain of vaginal tenderness or hematuria [2]. In Saudi Arabia, the results of one study done in the emergency department (ED) estimated the prevalence of UTI to be 9.9% of the total ED visits. The most prevalent and predominant causative pathogen was found to be *Escherichia coli* (*E. coli*), representing 45.83% of all pathogens isolated from the urine culture of adult patients [3]. UTIs account for the majority of emergency department visits and hospital admissions for which antibiotics are routinely prescribed. Among *E. coli* isolates from adults, high rates of resistance were found to ampicillin (58%) and co-trimoxazole (42%), which are antibiotics frequently prescribed for empirical treatment [3]. UTI treatment is also commonly associated with an inappropriate antibiotic prescription, with at least one type of error (46.2%) such as dose errors, duration errors, frequency errors, or inappropriate selection of antibiotic class [4]. This rising problem of antimicrobial resistance and inappropriate antibiotic prescription puts patients at risk and leads to elevated costs.

Comprehending the dynamics of the seasonality of UTIs can be beneficial to specify the causes and risk factors associated with infection and prevention. Previous studies showed that some organisms demonstrate seasonal manners in infections [5]. For example, bacterial meningitis infections occur frequently during winter [6,7]. On the other hand, *Salmonella* and *Campylobacter* infections are more common during summer [8,9]. Increased temperatures during summer can make people susceptible to dehydration [10]. Dehydration has been considered to increase the risk of UTIs by postponing bacterial removal from the urinary tract, reducing voiding frequency and rates of urine flow [11]. However, results from previous studies regarding the seasonality of UTIs are inconsistent. One study reported a high incidence in summer [12-16], while the other in winter [17].

Leukocyte esterase (LE) had a sensitivity of 87%, while the sensitivity of nitrites was 48% [18]. The specificity for leukocyte esterase and nitrate was 64% and 95%, respectively [18]. The positive predictive value (PPV) was 60% for LE and 85% for nitrites [18]. On the other hand, the negative predictive value (NPV) for LE was measured to be 89%, while the NPV for nitrites was 74% [18]. Nitrite has shown to be more useful in being an indication for the diagnosis of urinary tract infection (pooled diagnostic odds ratio (DOR): 11.3; 95% confidence interval (CI): 6.95-18.35) [19]. When it comes to symptomatic and asymptomatic UTIs, the use of a dipstick test does not give a clear distinction. A study assessed differences in symptomatic and asymptomatic patients above the age of 65 as regards the use of the dipstick test. It showed a slight increase in the sensitivity of symptomatic patients (73.7%) in comparison to asymptomatic patients (64.3%), while the specificity, PPV, and NPV were similar [20]. Moreover, the percentage of patients that demonstrated a positive dipstick while having a negative culture was 61% [20]. Therefore, using the dipstick test as the only indication of treatment could have yielded a high false estimate of UTI diagnosis and inappropriate use of antibiotics [2].

Antimicrobial resistance and antibiotic prescription errors around UTI treatment is a rising problem as it puts patients at risk and leads to elevated costs. Research on the seasonal patterns of UTIs is also limited and inconsistent. The use of the initial diagnostic methods for UTI (dipstick test) seems to not give a clear distinction between symptomatic and asymptomatic patients, further magnifying the effects of antibiotic resistance and which could lead to inappropriate diagnosis and treatment. Additionally, there is insufficient research on whether the presence of certain variables or comorbidities could be the cause of the inaccuracy of the results of diagnostic methods, as well as the lack of research on estimating the readmission rates among patients who were admitted with UTIs.

The aim of this study is to investigate the prevalence of urinary tract infection (UTI) diagnosed in patients admitted to the emergency department, investigate their seasonal patterns and diagnostic tests, and know the final diagnostic decision.

## Materials and methods

Study design, area, and settings

A retrospective cross-sectional study was conducted since the study is concerned with the prevalence of UTIs at a specific point in time. An estimate of the prevalence, diagnostic accuracy, and outcome of UTI among patients admitted through the ED of King Abdulaziz Medical City (KAMC) in Riyadh and its association with the season of the year were studied. This study was conducted in the ED and medical wards of KAMC.

Identification of study participants

The Raosoft sample size calculator was used in this study to calculate the sample size [21]. Keeping the confidence level at 95%, the margin of error at 5%, and an alpha of 0.05, the final sample size of the study included 400 patients with a primary admission diagnosis of UTI. Moreover, it was a non-probability consecutive sampling method. The study included patients admitted during specific months (January, April, July, and September) in 2019. The inclusion criteria were any Saudi patient being female or male over 18 years of age. The exclusion criteria were patients who are younger than 18. The outcome variables of this study were to recognize the effect of seasons on the prevalence of UTIs and show the variation between the prevalence of UTI admission diagnosis in ER and the final diagnostic decision, as well as to evaluate the sensitivity and specificity of the diagnostic methods used in the ED, for example, the dipstick test. According to a study that was done in Saudi Arabia, the prevalence of UTIs was 9.9% of the total ED visits. In the elderly and adults, they comprise around 14.6% of ED visits [4].

Data collection process

Data were obtained from the Hospital Information System BestCare of King Abdullah National Guard Hospital using the following search terms: urinary tract infection, pyelonephritis, cystitis, and urine culture. Only the charts for adults over 18 years of age admitted with UTI during the months of January, April, July, and September 2019 were included. The included records were reviewed for the demographic data, including sex and age in years, and urinalysis and microbiology data, including culture results and antibiotic sensitivity, Charlson Comorbidity Index (CCI) score, clinical presentation, vital signs, inpatient admission date, discharge date, length of hospital stay, and readmission within 30 days of discharge.

Data analysis

The variables of interest were arranged in a Microsoft Excel sheet (Microsoft Corp., Redmond, WA, USA) and analyzed using the JMP software (JMP Statistical Discovery LLC, Cary, NC, USA). Our categorical variables included the presence of UTI symptoms such as dysuria, frequency, urgency, rigors, and loin pain that were described as present, absent, or not mentioned, and the urinalysis and urine culture results that were represented as positive or negative. Numerical variables including temperature and vital signs were compared using the paired and independent t-tests. Since all the cases of the inclusion criteria covering different seasons and the variables were objective, biases were not significant.

Statistical analysis

The statistical software used to process data was the Statistical Package for Social Sciences software version 26 (IBM Corp., Armonk, NY, USA). Measuring the specificity and sensitivity was conducted through ROC curve analysis [22]. Categorical variables such as the presence of dysuria and urgency were presented as frequencies and percentages. It was evaluated using the chi-squared test for larger samples, while Fisher’s exact test was used for smaller samples [23,24]. In addition, numerical variables such as temperature and oxygen saturation were presented as means and standard deviations, and medians, and a comparison between them was done through paired and independent t-tests [25]. The possibility of relationship confounding between demographic variables and urinary tract infection was assessed using multiple logistics regression [26]. The tests were conducted assuring a p-value of <0.05 being the significance level and a 95% confidence interval (CI) for the prevalence rates. Complete insurance of confidentiality and anonymity was done through necessary means, including keeping the data in a secure location and the viewing limited to the investigators, in addition to not using the medical records number (MRN) or any information that could be an identifier for the patient.

## Results

Table 1 describes the characteristics of the patients during readmission. It was revealed that two patients had died during readmission. Similarly, 10.5% reported having readmission within 30 days of discharge due to UTI. The median days of readmission after discharge was 6.50 days (range: 2-20 days), and the most common diagnosis during readmission was pneumonia (2.5%), followed by UTI (2.2%) and cancer (1%).

**Table 1:** Characteristics of the patients during readmission (n = 315)

Variables	Number (%)
Did the patient die during readmission?	2 (0.60%)
Readmission within 30 days of discharge (for UTI)	33 (10.5%)
How long after discharge did the readmission occur (median (min-max))?	6.50 (2-20)
Primary readmission diagnosis	
No readmission	270 (85.7%)
Pneumonia	8 (2.5%)
UTI	7 (2.2%)
Heart failure	2 (0.60%)
Sepsis	2 (0.60%)
Cancer	3 (1%)
Kidney transplant status	2 (0.60%)
Hematoma	2 (0.60%)
Wound infection	1 (0.30%)
DVT	1 (0.30%)
Moderate protein-energy malnutrition	1 (0.30%)
Pulmonary embolism/fluid overload	1 (0.30%)
Hydronephrosis	1 (0.30%)
Neurogenic bladder	1 (0.30%)
Asthmatic attack	1 (0.30%)
Melena	1 (0.30%)
Fever with chills	1 (0.30%)
Epigastric pain	1 (0.30%)
Pleural effusion	1 (0.30%)
Multiple organ failure	1 (0.30%)
Ulcer of the penis	1 (0.30%)
Shortness of breath	1 (0.30%)
Urology surgery, cystoscopy, and cystourethrogram	1 (0.30%)
Neurogenic bladder	1 (0.30%)
Vomiting	1 (0.30%)
PEG tube change	1 (0.30%)
Fournier’s gangrene	1 (0.30%)

This study included 315 patients admitted with the diagnosis of UTI from a total of 3,011 ED visits. These included 125 (39.7%) male and 190 (60.3%) female. The mean age of the patients was 70.2 (SD: 18.1) years. The overall prevalence of patients admitted with the diagnosis of UTI was 10.50% of the total ED visits, while the prevalence of confirmed UTI cases was 67.93%. The highest prevalence was 13.30% in January, and the lowest was 8.50% in April. In July and September, the prevalences were 11.70% and 8.80%, respectively.

The median length of hospital stay was 3.5 days (range: 3-23 days), and 24.8% required isolation, while 2.9% transferred to intensive care unit (ICU) care during ER admission, and 4.1% were admitted to ICU on the first admission.

We used the chi-squared test to measure the relationship between previous admission due to UTI and the baseline characteristics of the patients (Table 2). Previous admission due to UTI was less common among patients with dysuria (p = 0.004), rigors (p = 0.046), and loin pain (p = 0.003), while it was more common among those who required isolation (p < 0.001) and those with readmission within 30 days of discharge (p = 0.007).

**Table 2:** Relationship between previous admission due to UTI and the baseline characteristics of the patients (n = 315) § P-value has been calculated using the chi-squared test. ** Significant at p < 0.05 level.

Factors	Previous admission due to UTI		p-value^§^
	Yes (number (%))^(n = 74)^	No (number (%))^(n = 241)^	
Gender			
Male	33 (44.6%)	92 (38.2%)	0.323
Female	41 (55.4%)	149 (61.8%)	
Dysuria	19 (25.7%)	113 (46.9%)	0.004**
Frequency	3 (4.1%)	20 (8.3%)	0.220
Urgency	2 (2.7%)	12 (5%)	0.514
Rigors	8 (10.8%)	51 (21.2%)	0.046**
Loin pain	5 (6.8%)	53 (22%)	0.003**
Urinalysis done in the ER	69 (93.2%)	224 (92.9%)	0.930
Leukocyte esterase	67 (90.5%)	10 (87.1%)	0.650
Nitrates	14 (18.9%)	56 (23.2%)	0.736
RBC/HPF	58 (78.4%)	178 (73.9%)	0.663
WCC/HPF	70 (94.6%)	215 (89.2%)	0.383
ICU referral in the ED	0	10 (4.1%)	0.075
Culture done in the ER	66 (89.2%)	210 (87.1%)	0.639
Midstream am urine culture	72 (97.3%)	221 (91.7%)	0.228
Antibiotic sensitivity	42 (56.8%)	158 (65.6%)	0.169
ESBL UTIs	22 (29.7%)	56 (23.2%)	0.258
Required isolation	31 (41.9%)	47 (19.5%)	<0.001**
Did the patient die during readmission?	1 (1.4%)	1 (0.40%)	0.375
Readmission within 30 days of discharge	14 (18.9%)	19 (7.9%)	0.007**
Went to ICU care within ER admission	2 (2.7%)	7 (2.9%)	0.927
Admitted to the ICU (first admission)	3 (4.1%)	10 (4.1%)	0.971

**Figure 1:**
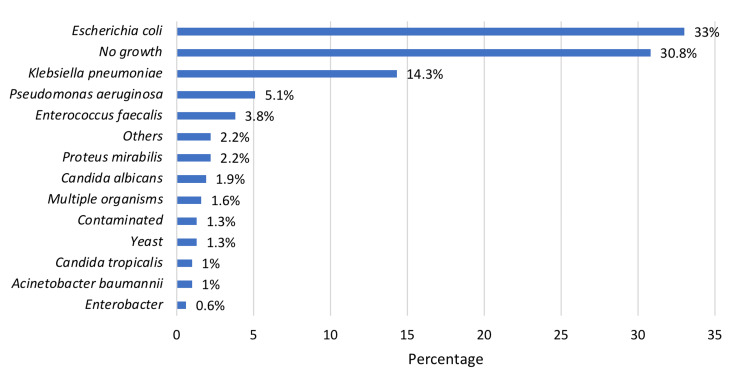
Bacteria isolated

Laboratory features including leukocyte esterase, urine nitrite, RBC/HPF, and WCC/HPF were positive in 87.9%, 22.2%, 74.9%, and 90.5%, respectively. Antibiotic sensitivity was detected among 63.5% of the patients. ESBL UTI constitute 24.8%, while VDRL UTI was detected in one patient. Figure 1 depicts the bacterial growth detected among patients. It can be observed that *E. coli* was the most common bacterial growth (33%), followed by *Klebsiella pneumoniae *(14.3%) and *Pseudomonas aeruginosa* (5.1%), whereas *Enterobacter* was the least detected (0.6%). The mean values of hemoglobin, WBC, platelets, MPV, and RDW were 108 gm/L, 8.32 × 10^9^/L, 316.5 × 10^9^/L, 7.68 fL, and 14.9%, respectively, while the mean values of serum sodium, serum potassium, serum urea, serum creatinine, serum bicarbonate, and serum albumin were 131.7 mmol/L, 4.83 mmol/L, 9.20 mmol/L, 131.3 umol/L, 23.1 mmol/L, and 33.5 g/L, respectively. In addition, the mean values of CRP, procalcitonin, SGOT, alkaline phosphatase, and lactic acid were 38.3 mg/L, 0.28 ng/mL, 29 U/L, 121.9 U/L, and 2.07 mmol/L, respectively.

A p-value cutoff point of 0.05 at 95% CI was used to determine statistical significance. The analyses measured the relationship between the sociodemographic and laboratory parameters in relation to previous readmission due to UTI using the chi-squared test (categorical variables) and independent sample t-test (continuous variables). Multivariate regression analysis had been also performed to determine the independent predictor associated with previous readmission due to UTI with odds ratio (OR) and 95% confidence interval.

When measuring the differences in the laboratory parameters during previous admission, it was observed that increasing age was associated with previous admission due to UTI (p = 0.034). Furthermore, the CCI score was significantly higher among those with previous admissions (p < 0.001), while the mean values of RDW (p < 0.001) and serum urea (p = 0.032) were significantly higher among patients with previous admission. On the other hand, the mean values of hemoglobin (p < 0.001) and serum albumin (p < 0.001) were significantly less among those patients with previous admission due to UTI (Table 3).

**Table 3:** Differences in the laboratory parameters and other continuous variables in previous admission for UTI (n = 315) § P-value has been calculated using the independent sample t-test. ** Significant at p < 0.05 level.

Factors	Previous admission due to UTI		p-value^§^
	Yes (number (%))^(n = 74)^	No (number (%))^(n = 241)^	
Age in years	74.1 ± 16.2	69 ± 18.5	0.034**
CCI	6.96 ± 3.04	5.32 ± 3.03	<0.001**
Temperature	37.2 ± 0.77	37.2 ± 0.82	0.990
SBP	128.7 ± 23.6	127.3 ± 22.2	0.652
DBP	67.8 ± 16.8	66.6 ± 15.4	0.597
Pulse rate	95.4 ± 24.4	90.1 ± 19.2	0.056
Oxygen saturation	96.9 ± 2.57	95.7 ± 6.45	0.132
Hemoglobin	108.2 ± 23.2	120.5 ± 20.3	<0.001**
WBC	11.1 ± 5.31	11.2 ± 5.33	0.948
Platelets	321.9 ± 135	290.7 ± 119.5	0.059
MPV	8.19 ± 1.38	8.17 ± 1.48	0.891
RDW	15.4 ± 2.22	14.2 ± 2.21	<0.001**
Serum sodium	135.3 ± 6.69	133.6 ± 6.94	0.067
Serum potassium	4.43 ± 0.68	4.53 ± 0.76	0.325
Serum urea	12.4 ± 7.79	10.3 ± 7.52	0.032**
Serum creatinine	153.2 ± 146.7	125.9 ± 105.2	0.078
Serum bicarbonate	24.5 ± 5.33	23.7 ± 5.33	0.447
Serum albumin	31.1 ± 5.88	34.2 ± 3.52	<0.001**
CRP	81.4 ± 68.9	83.8 ± 91.4	0.898
Procalcitonin	3.12 ± 12.2	4.33 ± 14.5	0.736
SGOT	30.4 ± 26.6	28.6 ± 23.4	0.599
Alkaline phosphatase	125.4 ± 73.3	120.7 ± 70.3	0.663
Lactic acid	1.74 ± 1.19	2.18 ± 3.83	0.388

In multivariate regression model, admission rate due to UTI was more associated with those who required isolation (AOR: 1.972; 95% CI: 1.005-3.871; p = 0.048), while admission rate was less associated on patients with dysuria (AOR: 0.482; 95% CI: 0.240-0.969; p = 0.041). In addition, the increase in admission was associated with a decrease in oxygen saturation (AOR: 0.878; 95% CI: 0.789-0.976; p = 0.017) and RDW (AOR: 0.853; 95% CI: 0.745-0.977; p = 0.022). On the other hand, age in years, CCI score, rigors, loin pain, readmission within 30 days of discharge, serum urea, and serum albumin were not the relevant factors of admission rate after adjustment to the regression model (Table 4).

**Table 4:** Multivariate regression analysis to determine the independent factor associated with previous admission due to UTI (n = 315) ** Significant at p < 0.05 level.

Factors	AOR	95% CI	p-value
Age in years	1.004	0.980-1.028	0.759
CCI	0.896	0.790-1.015	0.085
Dysuria	0.482	0.240-0.969	0.041**
Rigors	0.644	0.260-1.596	0.342
Loin pain	0.437	0.146-1.305	0.138
Required isolation	1.972	1.005-3.871	0.048**
Readmission within 30 days of discharge	1.738	0.713-4.233	0.224
Oxygen saturation	0.878	0.789-0.976	0.017**
RDW	0.853	0.745-0.977	0.022**
Serum urea	1.009	0.969-1.050	0.657
Serum albumin	1.032	0.971-1.095	0.311

Midstream urine cultures were taken among all patients. Urinalysis was done in the ER among 93%, while the urine culture was recorded among 87.6%.

In Table 5, the prevalence of culture in ER was significantly more in January (p = 0.023), while ESB UTIs were significantly less during September (p = 0.007). On the other hand, gender, dysuria, frequency, loin pain, rigors, urinalysis done in ER, antibiotic sensitivity, nitrates, leukocyte esterase, bacterial growth, and specific type of bacteria were not significantly different in four months (all: p > 0.05).

**Table 5:** Rates of urinalysis, cultures, and antibiotic sensitivity during admission in four months for the year 2019 (n = 315) * Patients without bacterial growth were excluded from the analysis. § P-value has been calculated using the chi-squared test. ** Significant at p < 0.05 level.

Factors	January (number (%))^(n = 90)^	April (number (%))^(n = 65)^	July (number (%))^(n = 87)^	September (number (%))^(n = 73)^	p-value^§^
Gender					
Male	42 (46.7%)	28 (43.1%)	32 (36.8%)	23 (31.5%)	0.213
Female	48 (53.3%)	37 (56.9%)	55 (63.2%)	50 (68.5%)	
Dysuria	43 (47.8%)	24 (36.9%)	38 (43.7%)	27 (37%)	0.220
Frequency	11 (12.2%)	2 (3.1%)	8 (9.2%)	2 (2.7%)	0.054
Loin pain	15 (16.7%)	13 (20%)	16 (18.4%)	14 (19.2%)	0.956
Rigors	21 (23.3%)	14 (21.5%)	13 (14.9%)	11 (15.1%)	0.384
Urgency	5 (5.6%)	0	7 (8%)	2 (2.7%)	0.073
ESBL UTIs	32 (35.6%)	14 (21.5%)	23 (26.4%)	9 (12.3%)	0.007**
Urinalysis in ER	88 (97.8%)	60 (92.3%)	78 (89.7%)	67 (91.8%)	0.181
Culture in ER	86 (95.6%)	53 (81.5%)	77 (88.5%)	60 (82.2%)	0.023**
Antibiotic sensitivity	60 (66.7%)	40 (61.5%)	57 (65.5%)	43 (58.9%)	0.726
Nitrates	21 (23.3%)	15 (23.1%)	19 (21.8%)	15 (20.5%)	0.621
Leukocyte esterase	82 (91.1%)	53 (81.5%)	79 (90.8%)	63 (86.3%)	0.069
Bacterial growth					
Yes	69 (76.7%)	42 (64.6%)	60 (69%)	47 (64.4%)	0.285
No	21 (23.3%)	23 (35.4%)	27 (31%)	26 (35.6%)	
Specific type of bacteria^(n = 218)*^					
Klebsiella pneumoniae	14 (20.3%)	5 (11.9%)	15 (25%)	11 (23.4%)	0.168
Escherichia coli	32 (46.4%)	25 (59.5%)	23 (38.3%)	24 (51.1%)	
Acinetobacter baumannii	1 (1.4%)	0	2 (3.3%)	0	
Candida albicans	4 (5.8%)	1 (2.4%)	0	1 (2.1%)	
Enterococcus faecalis	1 (1.4%)	3 (7.1%)	5 (8.3%)	3 (6.4%)	
Yeast	0	0	2 (3.3%)	2 (4.3%)	
Pseudomonas aeruginosa	5 (7.2%)	3 (7.1%)	7 (11.7%)	1 (2.1%)	
Proteus mirabilis	2 (2.9%)	0	1 (1.7%)	4 (8.5%)	
Multiple organisms	4 (5.8%)	0	0	1 (2.1%)	
Enterobacter	1 (1.4%)	0	1 (1.7%)	0	
Contaminated	1 (1.4%)	1 (2.4%)	2 (3.3%)	0	
Candida tropicalis	2 (2.9%)	1 (2.4%)	0	0	
Others	2 (2.9%)	3 (7.1%)	2 (3.3%)	0	

**Figure 2:**
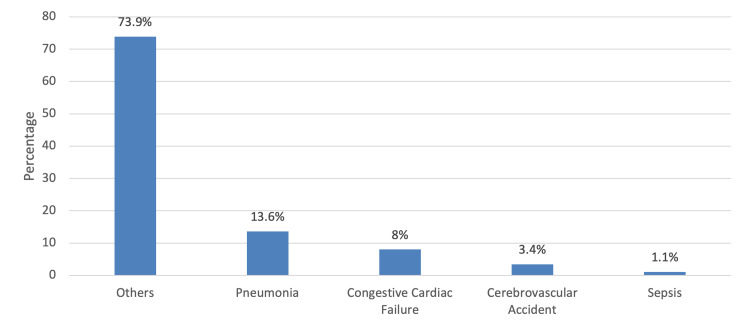
Reasons for previous admission over the last 90 days not related to UTI

The most common reason for admission over the last 90 days not related to UTI was pneumonia (13.6%), followed by congestive cardiac failure (8%) and cerebrovascular accident (3.4%), while sepsis was the least (1.1%) (Figure 2).

## Discussion

One of the study’s aims was to look into the prevalence of confirmed UTI cases among those admitted with an initial diagnosis of UTI as a total and through the months of January, April, July, and September. For those who were admitted through ED as a case of UTI prior to confirmation by culture, they constituted 10.50% of the total admission in the internal medicine department. The confirmation method was through calculating positive urinary cultures. The results of this study indicated a higher percentage of confirmed UTI cases in January, followed by July, September, and April. The total prevalence of the previous four months mentioned was 67.93%.

Out of our sample size of admitted patients through the emergency department with the diagnosis of UTI, *E. coli* is the main infectious organism that was found in all four months. This was followed by *K. pneumoniae* and then by *P. aeruginosa*, while the least prevalent of all bacteria isolated was *Enterobacter*. This corresponds to numerous studies such as one set in Al-Baha that reported that *E. coli* is the most commonly detected organism in community and hospital-acquired infections [27]. Another study published in 2018 that was conducted in Riyadh studying community-acquired UTIs in the emergency department showed a similar outcome of *E. coli* being the most common causative organism [4]. ESBL UTIs constituted 24.8% of the cases, while VDRL UTIs were detected in one patient. On the other hand, a study conducted in the USA yielded a prevalence of 5.9% of ESBL UTIs [28].

In this study, the percentage of female cases is higher than those of males. This was in line with another study that previously showed that 64.04% of the patients visiting the ER with a UTI were females [4]. The previous finding could be attributed to the difference in length between female and male urethra with the female having shorter urethras in addition to the presence of frequent vaginal colonization [29].

Dysuria is the most prevalent symptom, followed by rigors, closely followed by loin pain, then frequency, followed by urgency being the least prevalent. The percentage of cases of UTI referred to the intensive care unit in the emergency department was 3.2%, while those who were admitted to the ICU within the ER admission is 2.9%. On the other hand, cases that were admitted to the ICU during the first admission constitute 4.1% of the cases admitted as UTI diagnoses. In addition, 24.8% of the cases required isolation.

This study also assessed the association of the seasons with the prevalence of UTIs during the four months of January, April, July, and September. The results of the study showed that the number of cultures done in the ER before admitting the patients is the highest during January, therefore conveying a higher prevalence in January. The peak in January indicates a seasonal pattern linked to colder weather in Saudi Arabia, unlike the results of a study done in Greece where UTIs peaked in summer [13]. One study that was conducted in Finland on seasonality showed a peak in incidence during winter, which is similar to ours [30]. Although more cultures were positive in January compared to September, the result was only significant in January. There is no seasonal variation in antibiotic sensitivity, symptomatic UTIs, and urinalysis despite evidence of variation in cultures. In addition, our study showed no evidence of seasonal patterns related to gender, unlike the results of a study done in the United States, which showed that women are at high risk of developing UTIs during warmer weather [31]. In our opinion, the conflicting evidence regarding gender-related seasonality can be partly caused by the assessment of seasonal patterns in different geographical areas or inadequate methodology. Furthermore, some studies showed a relation of causative organisms to seasonality, for example, *Staphylococcus saprophyticus* showed a peak in incidence during late summer and early autumn [32,33]. Our study showed no relation of specific types of bacteria to the season of the year. In addition, the prevalence of the alarming and steadily increasing bacteria ESBL is less significant during September, but the underlying causes of fewer ESBL UTIs are unknown. Further research should shed light on this phenomenon by understanding the factors linked to the drop in ESBL UTIs because it may benefit in limiting trends of antimicrobial resistance and avoiding prolonged hospital stay and recurrence [34].

The estimated number of patients with dipstick urinalysis done in the ER was 293 (93%), with the rest of the patients having urinalysis obtained as outpatient or inpatient. Leukocyte esterase was positive in 87.9%, while nitrate was detected in 22.2%. This corresponds to the many studies on the diagnostic value of dipstick test, one being a Tunisian study that found leukocyte esterase (LE) sensitivity of 87% and specificity of 64%, with nitrate having a sensitivity of 48% and specificity of 95% [18]. Cultures done in the ER were recorded among 276 (87.6%) patients. Excluding contaminated cultures, 214 (67.93%) had positive bacterial cultures. The number of positive bacterial cultures compared to the overall number of patients admitted with UTI is striking and suggests that there is an overestimation of admissions with the diagnosis of UTI.

Out of the 315 patients, 23.5% had a previous admission over the previous 90 days for UTI. Out of these patients, the majority had one admission, followed by two admissions, and the least number of cases had more than two admissions over these 90 days. Using the chi-squared test, it was found that previous admissions due to UTIs were less prevalent among those patients with dysuria, rigors, and loin pain. In addition, the admission rate due to UTIs was less associated with patients with dysuria. This finding suggests that perhaps patients with recurrent admissions due to UTI are less likely to have urinary symptoms present during the time of presentation to the emergency department. On the other hand, the prevalence of previous admissions due to UTI was significantly higher in patients who required isolation and patients who got readmitted within 30 days of discharge. Age, Charlson Comorbidity Index score, RDW, and serum urea were also significantly higher in patients who were previously admitted due to UTI. On the contrary, hemoglobin and serum albumin were significantly less in patients of the same group. The most common diagnosis of any previous admission over the past 90 days not related to UTI was pneumonia, followed by congestive heart failure, cerebrovascular accidents, and sepsis.

In this study, we also analyzed the prevalence of readmission in patients after discharge with the diagnosis of UTI. It was found that 14.2% of patients get readmitted within 30 days of discharge, with the median number being seven days after discharge. Perhaps the most noteworthy finding is that out of these 45 patients, the majority had a readmission diagnosis of UTI (10.5% of all patients). After UTI, the most common diagnosis during readmission was pneumonia, followed by cancers. It was revealed that two patients had died during their readmission. Of note, previous admissions due to UTIs were more common in patients who were readmitted within 30 days of discharge. This high prevalence of both UTI and previous hospital admissions over the past 90 days and high prevalence of UTI readmissions has important implications on healthcare systems. Unplanned hospital readmissions greatly add to the annual healthcare cost and carry a significant burden. However, as of yet, no single intervention alone was found to be associated with a reduced risk of 30-day rehospitalization of patients [34,35]. Additional research is needed to further identify patient-level risk factors and hospital-level risk factors for the better management of patient discharge and reducing the burden of readmissions among patients.

This study’s limitations are that it was conducted in only one setting, the emergency department at King Abdullah Medical City, Riyadh, Saudi Arabia. Another limitation is the possibility of lost cases due to the definition of a positive urine culture being a bacterial growth of 105 CFU/mL with a single bacterium. In addition, the time period of this study was short (four months). Therefore, our recommendation is to extend the period of the study to more months yielding more generalized results.

## Conclusions

This study demonstrates that the prevalence of the diagnoses of UTIs among admissions from the ED was high despite some cultures being negative or contaminated. In addition, it displayed a seasonal pattern linked to the highest number of confirmed cases in January, while the lowest in April.
